# Ring finger protein 121 is a potent regulator of adeno-associated viral genome transcription

**DOI:** 10.1371/journal.ppat.1007988

**Published:** 2019-08-06

**Authors:** Victoria J. Madigan, Julianne A. Yuziuk, Anna M. Chiarella, Tyne O. Tyson, Rita M. Meganck, Zachary C. Elmore, Longping V. Tse, Nathaniel A. Hathaway, Aravind Asokan

**Affiliations:** 1 Curriculum in Genetics and Molecular Biology, University of North Carolina at Chapel Hill, Chapel Hill, NC, United States of America; 2 Gene Therapy Center, the University of North Carolina at Chapel Hill, Chapel Hill, NC, United States of America; 3 Department of Surgery, Duke University School of Medicine, Durham, NC, United States of America; 4 Division of Chemical Biology and Medicinal Chemistry, UNC Eshelman School of Pharmacy, Chapel Hill, NC, United States of America; 5 Department of Molecular Genetics & Microbiology, Duke University School of Medicine, Durham, NC, United States of America; The Children's Hospital of Philadelphia, UNITED STATES

## Abstract

Adeno-associated viruses (AAV) are Dependoparvoviruses that have shown promise as recombinant vectors for gene therapy. While infectious pathways of AAV are well studied, gaps remain in our understanding of host factors affecting vector genome expression. Here, we map the role of ring finger protein 121 (RNF121), an E3 ubiquitin ligase, as a key regulator of AAV genome transcription. CRISPR-mediated knockout of RNF121 (RNF121 KO) in different cells markedly decreased AAV transduction regardless of capsid serotype or vector dose. Recombinant AAV transduction is partially rescued by overexpressing RNF121, but not by co-infection with helper Adenovirus. Major steps in the AAV infectious pathway including cell surface binding, cellular uptake, nuclear entry, capsid uncoating and second strand synthesis are unaffected. While gene expression from transfected plasmids or AAV genomes is unaffected, mRNA synthesis from AAV capsid-associated genomes is markedly decreased in RNF121 KO cells. These observations were attributed to transcriptional arrest as corroborated by RNAPol-ChIP and mRNA half-life measurements. Although AAV capsid proteins do not appear to be direct substrates of RNF121, the catalytic domain of the E3 ligase appears essential. Inhibition of ubiquitin-proteasome pathways revealed that blocking Valosin Containing Protein (VCP/p97), which targets substrates to the proteasome, can selectively and completely restore AAV-mediated transgene expression in RNF121 KO cells. Expanding on this finding, transcriptomic and proteomic analysis revealed that the catalytic subunit of DNA PK (DNAPK-Cs), a known activator of VCP, is upregulated in RNF121 KO cells and that the DNA damage machinery is enriched at sites of stalled AAV genome transcription. We postulate that a network of RNF121, VCP and DNA damage response elements function together to regulate transcriptional silencing and/or activation of AAV vector genomes.

## Author summary

Recombinant AAV vectors are at the forefront of clinical gene therapy. There is a need to better understand the mechanisms dictating AAV transduction in the host. Here, we identify a network of host proteins involving RNF121, p97 and the DNA damage machinery as potent factors regulating AAV genome transcription. Our study sheds light on an understudied aspect of AAV biology with implications for gene therapy.

## Introduction

Adeno-associated viruses (AAVs) rely on co-infection of the host cell by a helper virus as well as several host factors for replication [1]. The 4.7kb single stranded DNA AAV genome contains two open reading frames flanked by two inverted terminal repeats (ITRs) packaged into an icosahedral capsid measuring 25nm in diameter [1,2]. The only required cis-packaging signal for generating recombinant AAV vector genomes are the two ITRs [1,2]. The AAV infectious cycle begins with binding of attachment factors on the cell surface, with different serotypes binding distinct glycan moieties, which have been linked to different tissue tropisms *in vivo* [1,3]. Following endocytic uptake, AAV traffics through endosomes and the golgi network to the nucleus [4]. Further, AAV is thought to enter the nucleus through nuclear pores with the capsid intact, only uncoating once inside this environment. The uncoated ssDNA AAV genome undergoes second-strand synthesis and is then transcribed, although the impact of host factors on the latter event remains poorly understood [5,6]. Understanding post-second strand synthesis events in AAV biology could shed light on AAV vector genome silencing that has been observed in gene therapy clinical trials [6,7].

Several high-throughput screening based studies have facilitated the discovery of previously unknown host factors that influence AAV transduction. For instance, high-throughput proteomic screens probing AAV2 and AAV8 host binding partners revealed a wealth of novel interactors, including CDK2/cyclinA kinase [8]. An siRNA screen elucidated host restriction factors involved in DNA damage response and cell cycle checkpoint activation [9]. A different siRNA screen demonstrated that the SUMOylation pathway restricts AAV transduction prior to nuclear entry and vector genome release [10]. In another siRNA screen, the U2 snRNP spliceosome was identified as a key host factor that limits transcription of the AAV vector transgene and specific components of the spliceosome validated as AAV capsid binding partners [11]. More recently, a haploid screen for essential AAV host factors uncovered the importance of a new universal AAV receptor, KIAA0319L or AAVR[12]. In the same study, amongst other hits for endosomal and Golgi related genes implicated as essential host factors, RNF121 or Ring Finger Protein 121 was identified as a potential AAV host factor.

In the current study, we dissect the previously uncharacterized role of RNF121 in AAV transduction. We utilize CRISPR knockout cell lines to demonstrate RNF121 depletion causes near ablation of AAV transgene expression, regardless of serotype, cell line, or vector dose. Interrogation of the importance of RNF121 at each step of the AAV infectious cycle is used to demonstrate that RNF121 is unnecessary for AAV cellular uptake, nuclear entry, uncoating, and production. A battery of RNA biology-focused assays including qRT-PCR, mRNA stability, and Chromatin Immunoprecipitation studies establish that RNF121 is essential for AAV genome transcription. Further, through interrogation of ubiquitination and degradation pathways, proteomics as well as transcriptome analysis, we discover that RNF121 is essential to prevent transcriptional arrest of AAV genomes potentially mediated by a complex network involving DNA-PKCs, VCP/p97 and the DNA damage machinery.

## Results

### RNF121 is an essential host factor for AAV transduction

**Fig 1:**
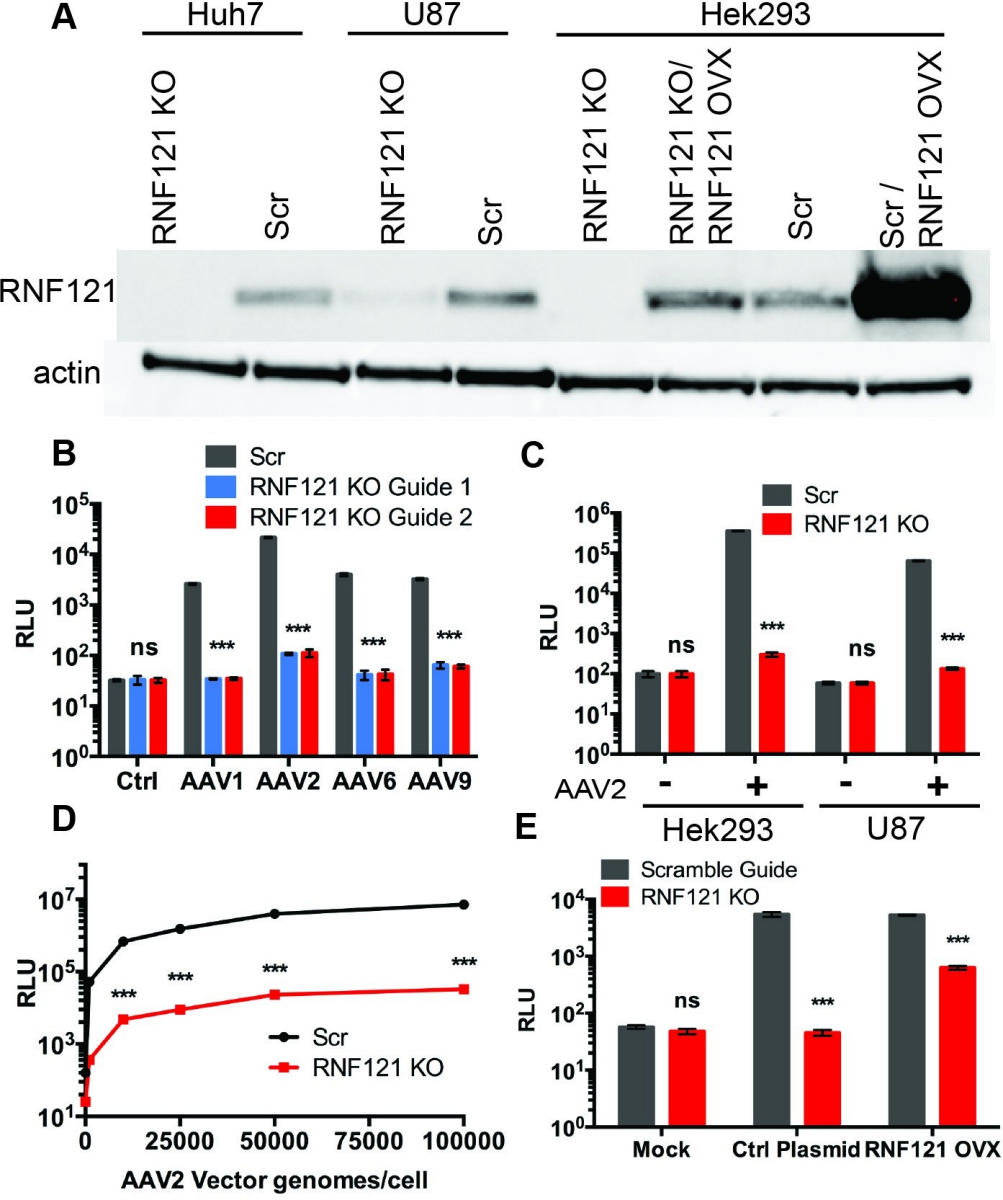
Effect of RNF121 knockout (KO) and overexpression (OVX) on AAV transduction. A, Western blot of whole cell lysates from Scr and RNF121 KO cell lines. B, Luciferase reporter expression of Scr and RNF121 KO Huh7 cells following transduction with AAV1 (10,000 vg/cell), AAV2 (5,000 vg/cell), AAV6 (10,000 vg/cell), and AAV9 (20,000 vg/cell). C, Luciferase reporter expression of Scr and RNF121 KO in HEK293 and U87 cell lines following transduction with AAV2. D, Luciferase reporter signal with increasing dose of AAV2 on Scr and RNF121 KO Huh7 cells. E, Luciferase reporter signal of Scr and RNF121 KO HEK293 cells following transfection with a control plasmid or plasmid containing RNF121 cDNA. A two-tailed unpaired t test was used unless otherwise indicated, with *, p<0.05; **, p<0.01; ***, p<0.005.

CRISPR guide RNAs targeting RNF121, as well as a nontargeting Scrambled guide control, were cloned into the LentiCrisprV2 backbone and used to produce recombinant lentivirus. We transduced Huh7 hepatocarcinoma cells with these constructs and selected with puromycin to produce stable knockout lines. Clonal lines were isolated via serial dilution and knockout validated via western blot and high-throughput sequencing of indel sites (Fig 1A, S1 Fig). RNF121 knockout (RNF121 KO) lines show a highly significant, 2-log-fold knockdown of AAV2-luciferase transgene expression in comparison to non-targeting (Scr) control line (Fig 1B). This phenotype was recapitulated in the HEK293 human embryonic kidney and U87 glioblastoma cell lines, which were chosen in order to interrogate the consequences of RNF121 KO in divergent cell lines derived from other tissues (Fig 1C, S1 Fig). RNF121 KO mediated decrease in transduction was observed for multiple serotypes tested, including AAV1, AAV6, and AAV9 (Fig 1B). RNF121 KO and Scr Huh7 cells were also transduced with AAV2-luciferase at a range of 100–100,000 vector genomes per cell. A 2-log decrease in transgene expression was observed independent of vector dose (Fig 1D). We next generated an RNF121 cDNA plasmid and validated it by western blot after transfection (Fig 1A). Transfection of RNF121 cDNA in Scr HEK293 cells did not enhance transduction over an empty vector control, suggesting that RNF121 is not typically a limiting factor in tissue culture models (Fig 1E). However, overexpression partially restored AAV infectivity to previously resistant RNF121 KO cells (Fig 1E).

### RNF121 does not affect adenoviral transduction or adenoviral helper functions

**Fig 2:**
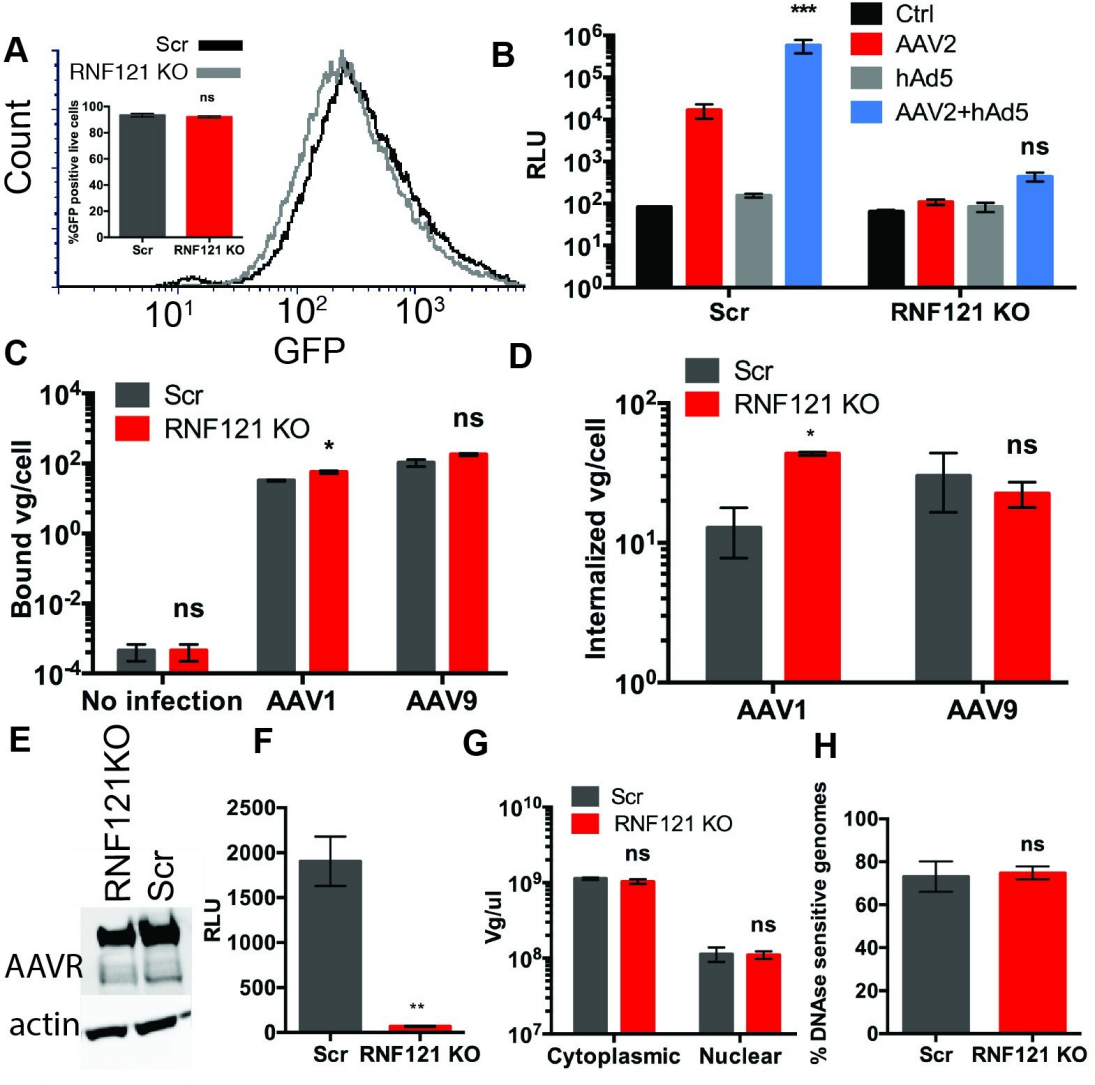
Effect of RNF121 KO on helper adenovirus infection and early steps in the AAV infectious pathway. A, Flow cytometric analysis and quantification of Scr and RNF121 KO Huh7 cells transduced with recombinant human Ad5-GFP (0.1 vg/cell). B, Luciferase reporter expression of Scr and RNF121 KO Huh7 cells following AAV2 transduction, with or without wild type hAd5 coinfection. C, Binding and D, Uptake of AAV1 and AAV9 capsids by Scr and RNF121 KO Huh7 cells. E, Immunoblot of AAVR expression relative to actin in Scr and RNF121 KO cells. F, Reporter expression of AAV4 (AAVR-independent serotype) in Scr and RNF121 KO cells. G, Quantitation of AAV vector genomes in cytoplasmic and nuclear fractions of Scr and RNF121 KO Huh7 cells following transduction with AAV2. H, Uncoating of vector genomes, quantified by qPCR of DNAse resistant genomes relative to total vector genomes in nuclear extract from Scr and RNF121 KO Huh7 cells. A two-tailed unpaired t test was used unless otherwise indicated, with *, p<0.05; **, p<0.01; ***, p<0.005.

Given the role of Adenovirus as a helper virus for AAV replication, we investigated whether RNF121 was an essential host factor for Adenovirus transduction. We transduced RNF121 KO Huh7 with recombinant human Adenovirus 5 expressing GFP (hAd5-GFP)[13]. Flow cytometric analysis demonstrated similar GFP expression in RNF121 KO cells relative to non-targeting control after hAd5-GFP transduction (Fig 2A). Because Adenovirus enhances AAV transduction through more efficient endosomal trafficking and transcriptional activation [14–16], we postulated that helper virus genes could rescue AAV transduction in RNF121 KO cells. However, coinfection of wild-type human adenovirus 5 with AAV2 luciferase did not fully restore AAV transduction in the Huh7 knockout line, though coinfection did significantly enhance transduction in Scr cells, as determined by two-way ANOVA analysis (Fig 2B).

### Titers and infectivity of AAV vectors generated from RNF121 KO cells are unaffected

To further investigate the role of RNF121 in the later steps in the AAV infectious pathway, we utilized Scr control and RNF121 KO HEK293 to produce AAV2 vectors packaging a luciferase transgene cassette by triple plasmid transfection [17]. We found no difference in AAV vector yield between the two cell lines, and the vectors produced displayed similar infectivity as well (S2A and S2B Fig).

### RNF121 does not play a role in cellular entry of AAV

We interrogated the different steps in the infectious pathway of AAV in RNF121 KO cells. Viral binding and uptake assays were performed as previously described [17]. Briefly, cells were pre-chilled and incubated with virus at 4°C to prevent cellular uptake. Following three PBS washes, vector genome DNA was harvested to quantify binding. To monitor cellular uptake, cells were moved to 37°C for one hour, trypsinized to remove bound, but not internalized virions, washed, then vector genome DNA harvested for quantitation. As seen in Fig 2C and 2D, binding and uptake assays demonstrate no defect in these early steps of the AAV infectious pathway in RNF121 KO cells, regardless of AAV serotype. RNF121 KO cells demonstrated a slight yet significant increase in binding and uptake of AAV1 (Fig 2C and 2D). However, this modest change is unlikely to account for the large decrease observed in transduction efficiency.

Given the previously characterized importance of AAVR in AAV cellular entry, we then investigated AAVR expression in RNF21KO cells via western blot [12]. Whole cell lysate from Scr and RNF121 KO HEK293 had similar levels of AAVR (Fig 2E). Characterization of the distinct interaction of each AAV serotype with AAVR has demonstrated that some AAVs, e.g., AAV4 infect cells through an AAVR-independent pathway [18]. To investigate whether AAVR independence can rescue transduction in the absence of RNF121, we infected RNF121 KO cells with AAV4. However, AAV4 displayed a transduction defective phenotype in RNF121 KO cells mirroring that of other serotypes (Fig 2F).

Next, cytoplasmic and nuclear fractions were harvested from RNF121 KO and control Scr cells following AAV transduction. AAVs have previously been characterized to enter the nucleus through nuclear pores with the capsid intact, only uncoating following nuclear entry [19,20]. The distribution of viral genomes was similar for both cell lines, indicating that both cytoplasmic and nuclear entry is not affected in the context of RNF121 KO (Fig 2G). Further, AAV uncoating was interrogated as previously described [21]. Briefly, nuclear extracts were harvested from Scr and RNF121 KO cells transduced with AAV. One half of each extract was subject to Benzonase treatment, whereby uncoated genomes would be degraded, while the second aliquot of nuclear extract was untreated. Both fractions were subject to qPCR to assess the relative number of vector genomes. No statistically significant difference was observed between the percentage of uncoated vector genomes in RNF121 KO vs Scr cells, and the percentage of uncoated vector genomes was consistent with previous publications (Fig 2H) [21].

### RNF121 KO is not rescued upon bypassing second-strand DNA synthesis

**Fig 3:**
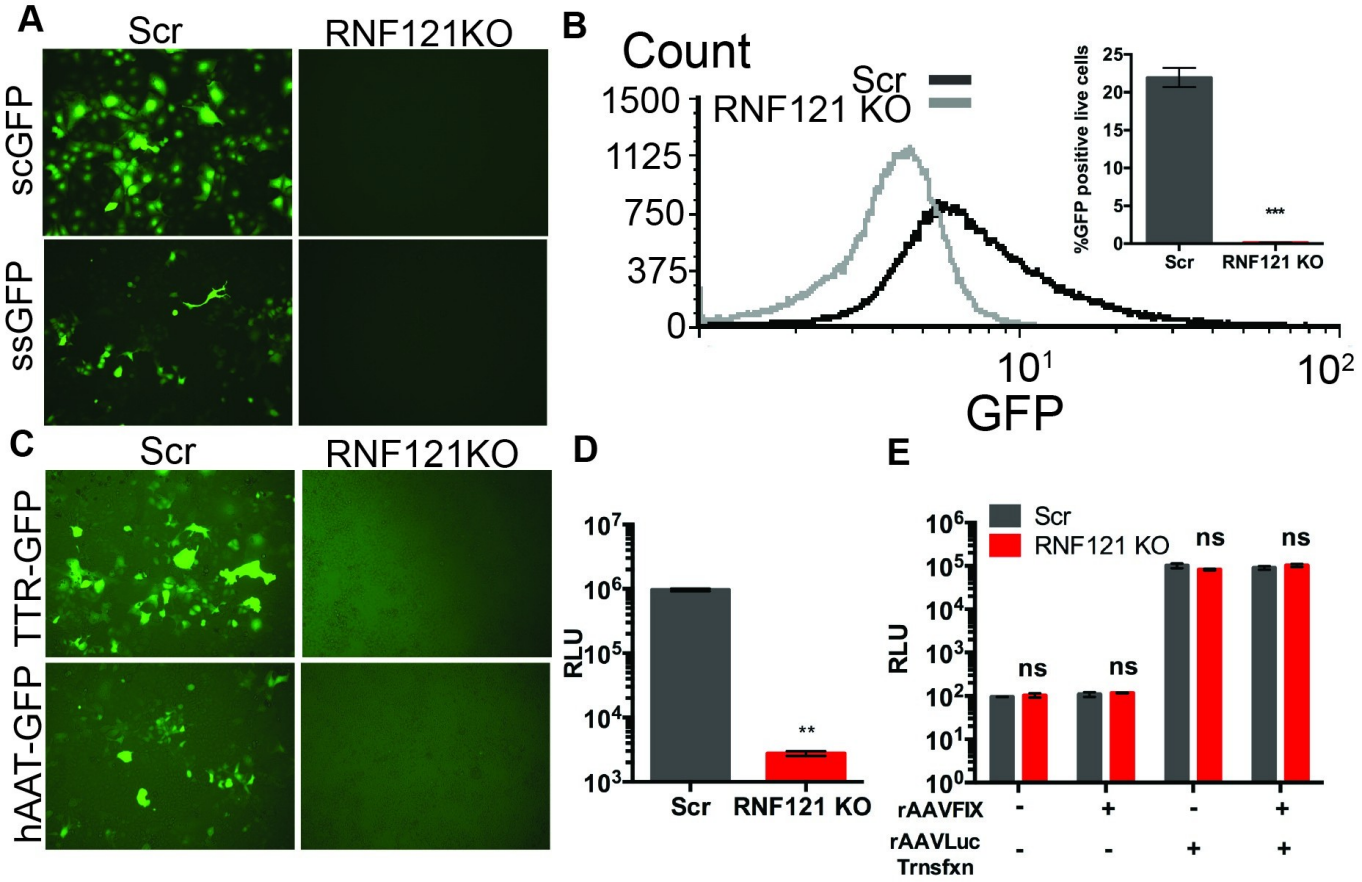
Effect of AAV genome architecture and promoter elements on transduction in RNF121 KO cells. A, GFP expression in Scr and RNF121 KO Huh7 cells transduced with self-complementary (scGFP; 2000 vg/cell) and single-stranded (ssGFP; 10,000 vg/cell) AAV2 vectrors. B, Flow cytometric quantitation of GFP positive cells in the AAV2-scGFP treated group. C, GFP expression in Scr and RNF121 KO Huh7 cells with AAV2 vectors packaging scGFP cassettes driven by Transthyretin (TTR) and human Alpha 1 Antitrypsin (hAAT) promoters. D, Luciferase expression in Scr and RNF121 KO HEK293 cells with AAV2 vectors packaging ssLuciferase cassettes driven by the CMV promoter. E, Transfection of Scr and RNF121 KO HEK293 cells with vector genomic DNA extracted from AAV2 Luciferase preparations, in the presence or absence of AAV8 capsids packaging a Factor IX cassette (100,000 vg/cell). A two-tailed unpaired t test was used unless otherwise indicated, with *, p<0.05; **, p<0.01; ***, p<0.005.

Second-strand synthesis of the ssDNA AAV genome typically represents a rate limiting step in AAV gene expression; however, this step can be circumvented with the use of self-complementary vectors, which contain an inverted repeated genome that can self-anneal without host DNA synthesis machinery [1,22]. Transduction of Scr and RNF121 KO Huh7 cells with single-stranded and self-complementary AAV2-GFP vectors demonstrated no measureable GFP transgene expression with either vector in RNF121 KO cells (Fig 3A). To further probe this step we transduced control and RNF121 KO cells with a self-complementary GFP vector, and analyzed GFP expression via flow cytometry. Flow cytometric quantification of rAAV2-scGFP expression confirmed our observed ablated expression of self-complementary vectors (Fig 3B), suggesting RNF121 KO cannot be rescued with vectors that can circumvent second-strand synthesis.

### Different promoter/intronic elements in the AAV genome are universally affected by RNF121 KO

To assess the effect of different promoter elements on AAV genome expression in RNF121 KO, we transduced Scr and RNF121 KO Huh7 with AAV2-GFP cassettes driven by the liver specific promoters hAAT and TTR and the MVM intron. We found GFP transgene driven by these promoters was also deficient in RNF121 KO cells (Fig 3C). Additionally, we transduced Scr and RNF121 KO HEK293 with rAAV2-CMV-Luciferase, finding CMV-Luciferase transduction recapitulated the phenotype seen with CBA driven vectors (Fig 3D). These data confirmed that altering rAAV promoter cassettes was not sufficient to rescue transduction.

### The AAV capsid is essential for the transduction defective phenotype in RNF121 KO cells

To determine whether AAV genomes were affected in RNF21 KO cells in a capsid-independent manner, we purified single-stranded, vector genomic DNA from AAV capsids packaging the luciferase transgene flanked by ITRs, and transfected capsid-free vector genomes into RNF121 KO cells. No effect in transgene expression relative to Scr was seen (Fig 3E). To determine whether AAV capsids in *trans* were capable of repressing transduction in the context of RNF121 KO, KO and control cells were transfected with the vector genome, following which with the cells were infected with AAV capsids packaging a different (human factor FIX) transgene cassette. Again, no change in luciferase transgene expression was observed, demonstrating that capsid presence in *trans* does not repress expression of transfected AAV genome in RNF121 KO cells (Fig 3E). These results suggest that RNF121 KO specifically affects gene expression from vector genomes packaged into AAV capsids.

### RNF121 is essential for transcription of both recombinant and wild type AAV genomes

**Fig 4:**
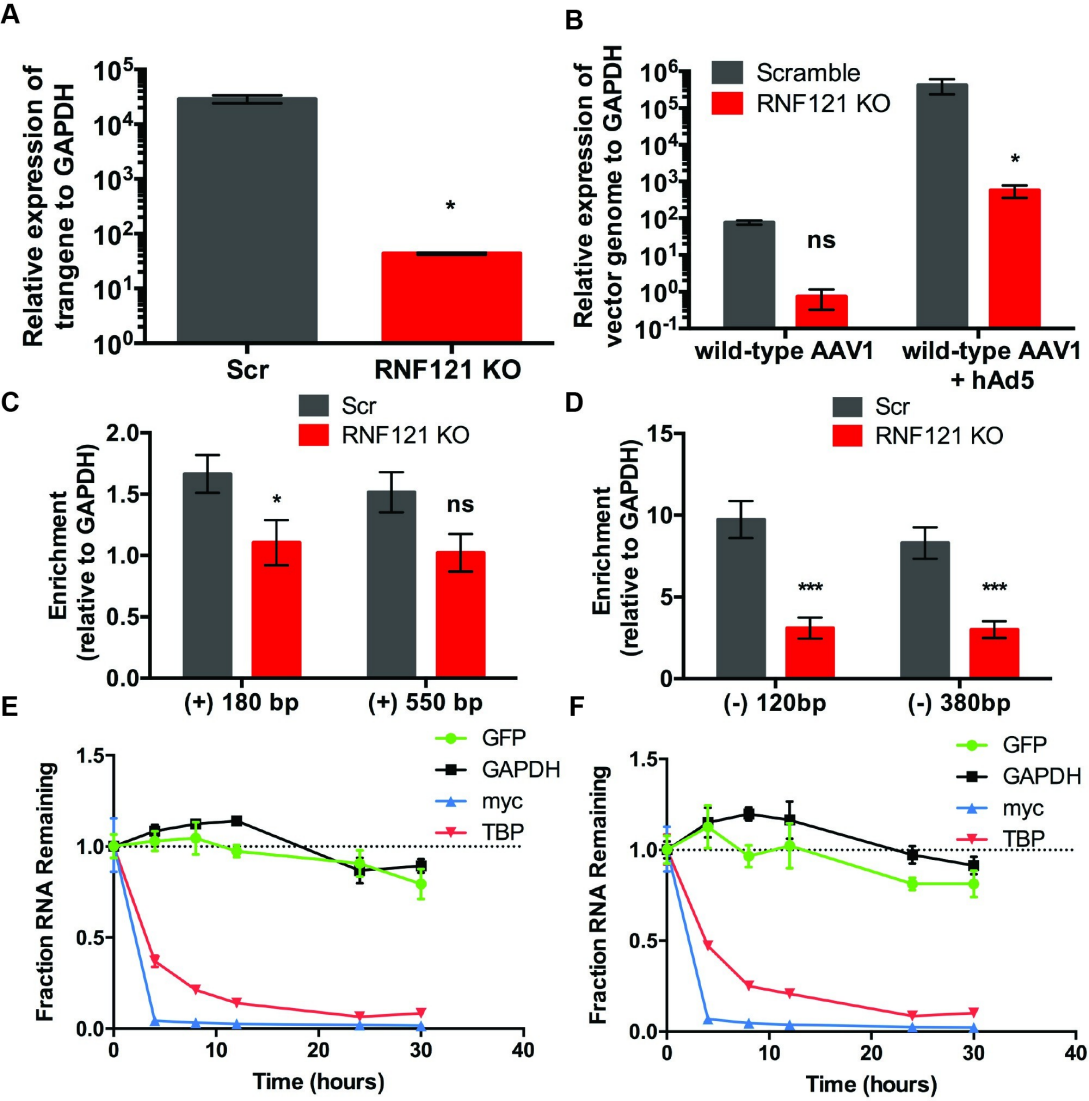
Effect of RNF121 KO on AAV genome transcription. A, Quantitative RT-PCR of AAV GFP genome expression relative to GAPDH housekeeping control in Scr and RNF121 KO Huh7 cells. B, qRT-PCR of wtAAV Rep gene expression relative to GAPDH housekeeping control in Scr and RNF121 KO Huh7 cells with and without wild type hAd5 coinfection. Chromatin immunoprecipitation of Scr and RNF121 KO HEK293 cells transduced with AAV2-Luciferase with antibodies against H3K27 acetylation (C) and serine 5 phosphorylated RNA polymerase II (D), followed by qPCR using primers against the Luciferase transgene and GAPDH. Actinomycin D mRNA stability time course profiles for Scr, 10,000 vg/cell (E) and RNF121 KO, 100,000 vg/cell (F) HEK293 cells following transduction with AAV2-GFP. A two-tailed unpaired t test was used unless otherwise indicated, with *, p<0.05; **, p<0.01; ***, p<0.005.

We then evaluated the impact of RNF121 KO on transcription of AAV vector genomes. Scr and RNF121 KO cells were transduced with AAV2-GFP, and transgene-derived mRNA levels were quantitated by qRT-PCR relative to mRNA levels of the housekeeping gene, GAPDH. As shown in Fig 4A, transgene mRNA was reduced by over two orders of magnitude in RNF121 KO cells relative to Scr control. Further, we also infected Scr and RNF121 KO cells with AAV capsids packaging the endogenous wild type genome (wtAAV) in the presence and absence of human Ad5 to assess the impact of RNF121 on transcription of wtAAV genes. A robust decrease in wtAAV genomic mRNA was observed as well, measured using qRT-PCR with primers against the AAV *Rep* gene (Fig 4B), which was determined to be significant via two-way ANOVA in the case of wtAAV and adenoviral coinfection.

We then carried out a chromatin immunoprecipitation (ChIP) assay to determine whether AAV vector genomes were differentially modified in the context of RNF121 KO. Following transduction of Scr and RNF121 KO HEK293 with AAV2-Luc for 48 hours, we analyzed epigenetic marks near the transcriptional start site (TSS) of the AAV vector genome. First, we probed H3K27 acetylation (H3K27ac), a marker of transcriptional activation. Previously, inhibition of histone deacetylases has been shown to enhance AAV genome expression, suggesting that acetylation may be important for transcriptional potency of AAV genomes [23,24]. At 180 bp downstream of the TSS, Scr cells had a 1.51 fold increase in H3K27ac relative to RNF121 KO, while 550 bp downstream of the TSS Scr cells showed a 1.48 fold increase in H3K27ac (Fig 4C). However, it is unlikely that this modest difference is a major contributor to the robust transcriptional phenotype of RNF121 KO. We also investigated changes in the recruitment of transcriptional machinery using an antibody against serine 5 phosphorylated RNA polymerase II (phosphoRNApolII), an activated form of RNA polymerase [25]. AAV genomes demonstrated statistically significant 3-fold depletion of phoshpoRNApolII both 120 and 380 bp upstream of the TSS in RNF121 KO cells relative to Scr control (Fig 4D). As shown in S3A and S3B Fig, we did not observe altered recruitment of these modifications in the host GAPDH gene, suggesting that RNF121 KO specifically reduces RNA pol II association with the AAV genome.

Because RNF121 KO profoundly affects levels of AAV genomic mRNA, we also assayed mRNA stability in the absence of RNF121 to determine whether the lack of AAV mRNA in RNF121 KO is due in part to mRNA degradation. We performed an Actinomycin D time course to assay mRNA stability, transducing RNF121 KO cells with 100,000 viral genomes/cell to achieve modest transcription of the AAV genome relative to a lower dose (10,000 vg/cell) on Scr cells. Briefly, qRT-PCR analysis was carried out targeting the AAV vector genome (GFP transgene), as well as GAPDH, TATA-Binding Protein (TBP), and c-myc, three host genes with decreasing mRNA half-life (Fig 4E and 4F). Scr and RNF121 KO cells demonstrated similar sustained presence of GAPDH, a highly stable transcript, as well as similar depletion of c-myc and TBP transcripts, which are known to be quickly degraded [26]. Approximately 80% of AAV GFP mRNA was present at 30hrs post-infection in both Scr and RNF121 KO cells indicating that reduced stability of the AAV mRNA transcript cannot explain the large decrease in steady-state AAV mRNA levels seen in RNF121 KO cells. These data are consistent with the AAV genome having a deficit in transcription in RNF121 KO, rather than altered kinetics or mRNA half-life.

### The E3 Ligase activity of RNF121 is essential, but may not act directly on AAV capsids

**Fig 5:**
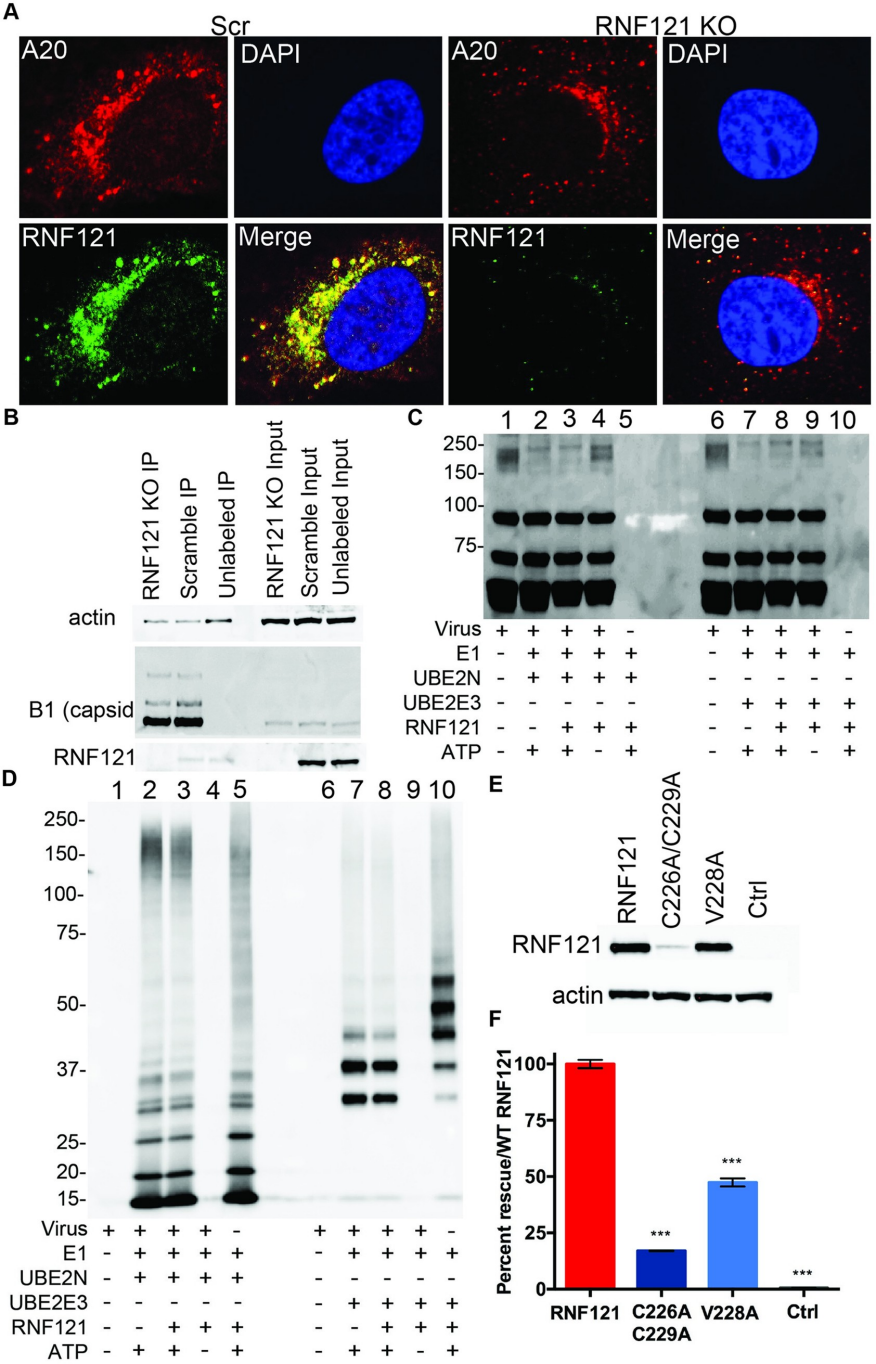
Effect of RNF121 KO on AAV capsid localization and ubiquitination. A, Representative images from confocal microscopy of Scr and RNF121 KO Huh7 cells at 12 hours post-transduction with AAV2, immunostaining for AAV2 Capsid (A20, Red), RNF121 (green), nuclei (DAPI/blue), and merged. B, Immunoprecipitation (IP) of biotinylated AAV2 capsids and immunoblotting of input and pull down material for actin, capsid, and RNF121. In vitro ubiquitination of capsids followed by immunoblotting for VP1,2,3 proteins (C) and ubiquitin (D). E, Immunoblot of RNF121 expression from catalytically dead mutants (C226A/C229A; V228A). F, AAV2-Luciferase transduction of RNF121 KO HEK293 cells following transfection of catalytically dead mutants. A two-tailed unpaired t test was used unless otherwise indicated, with *, p<0.05; **, p<0.01; ***, p<0.005.

Scr and RNF121 KO cells were transduced with AAV2 vectors and subject to confocal microscopy to assess immunofluorescence-based colocalization, probing for both the AAV capsid (via A20 antibody) and RNF121. A20 binds intact AAV capsids, typically localizing around the perinuclear space as AAVs traffic through the endosome and Golgi apparatus [27]. Intracellular localization of AAV capsids was similar between the Scr and RNF121 KO cell lines and demonstrated a perinuclear pattern (Fig 5A). Further, RNF121 demonstrated a perinuclear pattern in Scr cells, consistent with earlier reports and as expected, did not show any fluorescent signal in KO cells. (Fig 5A) [28,29]. RNF121 also demonstrated marked colocalization with AAV capsids in Scr cells (Fig 5A).

To assess whether RNF121 may be directly interacting with AAV capsids, AAV2 capsids were biotinylated as previously described [19], and RNF121 KO and Scr cells transduced with biotinylated vector were subject to immunoprecipitation (IP) using an anti-biotin antibody (Fig 5B). Blotting of input whole cell lysate confirmed no difference in capsid protein in transduced Scr vs RNF121 KO cells, and anti-biotin immunoprecipitation of these lysates successfully pulled down capsid in both Scr and RNF121 KO samples relative to an unlabeled virus control (Fig 5B). However, we were unable to pull down RNF121 with the capsid above background levels (Fig 5B).

Because many E3 ligases interact transiently with their substrates, we sought alternative methods to determine whether RNF121 ubiquitinates AAV capsid proteins. Using recombinant human RNF121, purified AAV capsids, E1 activating and E2 conjugating enzymes (UBE2N, UBE2E3), we performed cell-free *in vitro* ubiquitination assays of AAV capsids. We probed capsids incubated with these enzymes with antibodies against denatured AAV capsid proteins (VP1/2/3) as well as ubiquitin (Fig 5C and 5D). While we were able to detect AAV capsid VP ubiquitination using an anti- ubiquitin antibody and higher molecular weight bands via anti-VP antibody upon treatment with UBE2N/2E3 E2 conjugating enzymes, ubiquitination was still detectable in the absence of recombinant RNF121 E3 ligase. The addition of recombinant RNF121 failed to alter the presence of high molecular weight AAV capsid bands and ubiquitin signal (Fig 5C and 5D, lanes 2 vs 3 and 7 vs 8), with E1 and E2 alone producing the same ubiquitination pattern. Therefore, we hypothesized that the capsid proteins VP1/2/3 may not be direct substrates of RNF121.

To understand whether the E3 ligase activity of RNF121 is essential for AAV genome transcription in the cellular context, we generated two catalytic mutants of RNF121; C226A/C229A, described previously, as well as V228A, based on bioinformatic prediction of the RNF121 catalytic site [30]. These plasmids were transfected into RNF121 KO HEK293 cells and assessed for their ability to rescue AAV2-Luciferase expression relative to the WT RNF121 plasmid construct or a negative control plasmid (Fig 5E and 5F). C226A/C229A failed to rescue, but also did not express at wild-type levels; however, V228A RNF121, which was expressed at the same level of WT RNF121 also failed to fully rescue AAV genome expression. The latter mutant only achieved under 50% transduction as seen when rescuing the defective phenotype with the WT RNF121 plasmid. (Fig 5E and 5F).

### Pharmacological interrogation of the ubiquitin-proteasome pathway reveals a critical role for VCP/p97 in RNF121 KO cells

**Fig 6:**
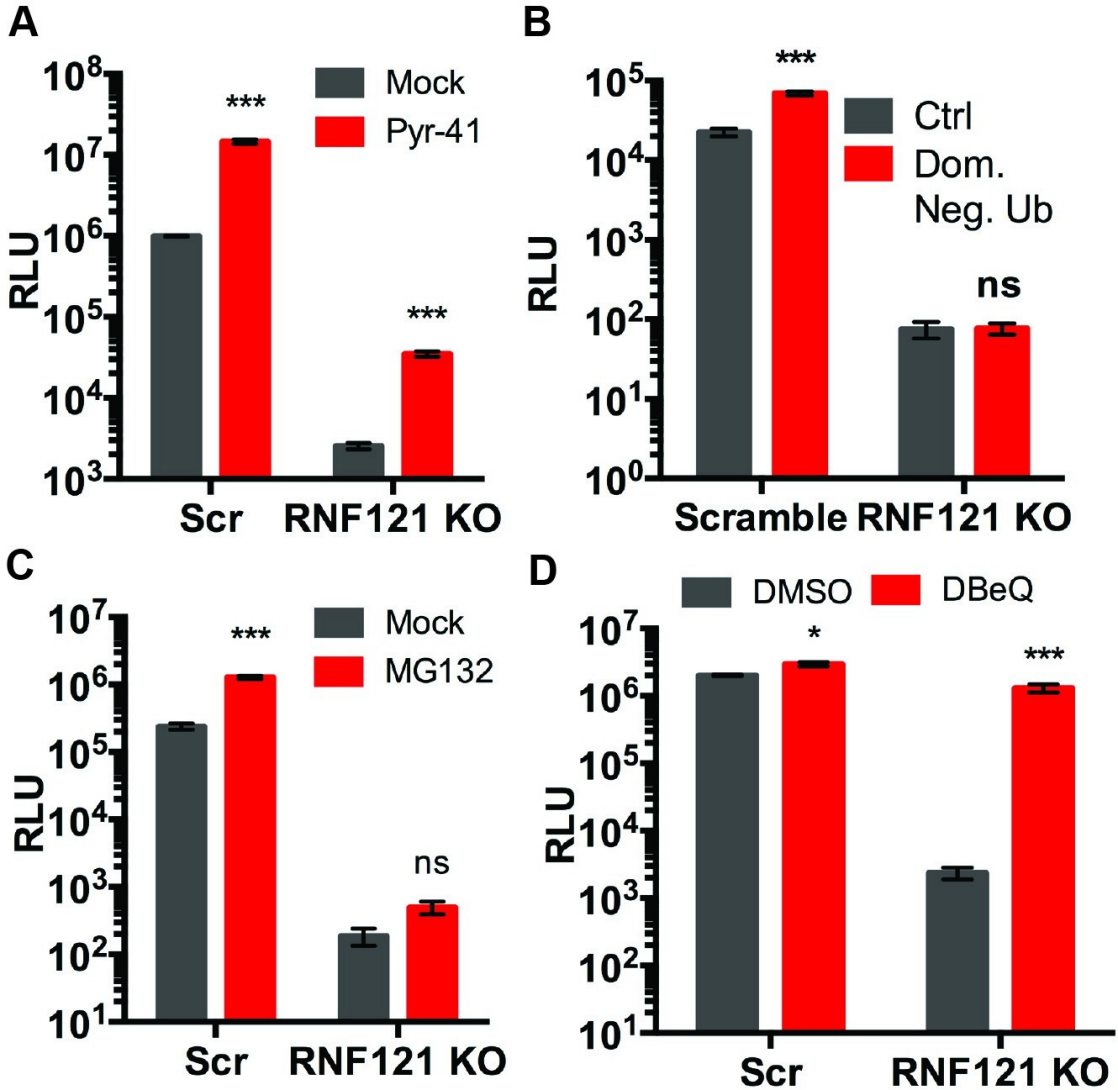
Pharmacological interrogation of ubiquitin-proteasome pathways in RNF121 KO cells. A, AAV2-Luciferase expression of Scr and RNF121 KO Huh7 cells treated with PYR-41 (50uM). B, AAV2-Luciferase expression following transfection of dominant negative ubiquitin construct. C, AAV2-Luciferase expression of Scr and RNF121 KO Huh7 cells treated with MG132 (10uM). D, AAV2-Luciferase expression following treatment of Scr and RNF121 KO cells with DBeQ (10uM). Luciferase transgene expression was assessed at 24hrs post-transduction. A two-tailed unpaired t test was used unless otherwise indicated, with *, p<0.05; **, p<0.01; ***, p<0.005.

First, since E3 ubiquitin ligases such as RNF121, typically act in conjunction with E1 activating and E2 conjugating enzymes, we evaluated PYR-41, a pan-inhibitor of E1 activating enzymes [31,32] (Fig 6A). PYR-41 treatment increased transduction in both Scr and RNF121 KO by roughly one order of magnitude. Further, we blocked ubiquitination by transfecting a dominant negative ubiquitin construct, which prevents ubiquitin chain elongation by adding a terminal ubiquitin without lysine residues [33]. As shown in Fig 6B, transfection of the dominant negative ubiquitin increased transduction in Scr cells by over 3-fold, while transduction in RNF121 KO cells was not affected. Thus, while ubiquitination status might affect AAV transduction in general, blocking this process did not rescue the RNF121 KO phenotype.

Next, proteasomal degradation of AAV particles is known to inhibit transduction, and treatment of cells with proteasome inhibitor MG132 has been previously shown to increase AAV transduction [34]. We treated both Scr and RNF121 KO cells with MG132 prior to transduction (Fig 6C). While Scr cells demonstrated a 5-fold increase in transduction, RNF121 KO cells demonstrated a modest increase of approximately 2-fold, which did not rescue the ablated expression caused by RNF121 KO (Fig 6C).

We next investigated the potential role of the segregase/unfoldase, Valosin containing protein (VCP), also known as p97, a AAA+ ATPase known to target substrates to ubiquitin-proteasome pathways and orchestrate the DNA Damage response [35–37]. Blocking VCP with the selective and reversible inhibitor, DBeQ did not substantially alter transduction in Scr cells, but resulted in over 500 fold enhancement of transduction in RNF121 KO cells (Fig 6D). This increase in transduction selectively and almost completely rescued the RNF121 KO phenotype (Fig 6D).

### Transcriptomic analysis reveals upregulation of DNA-Protein Kinase (DNA-PKCs) in RNF121 KO

**Fig 7:**
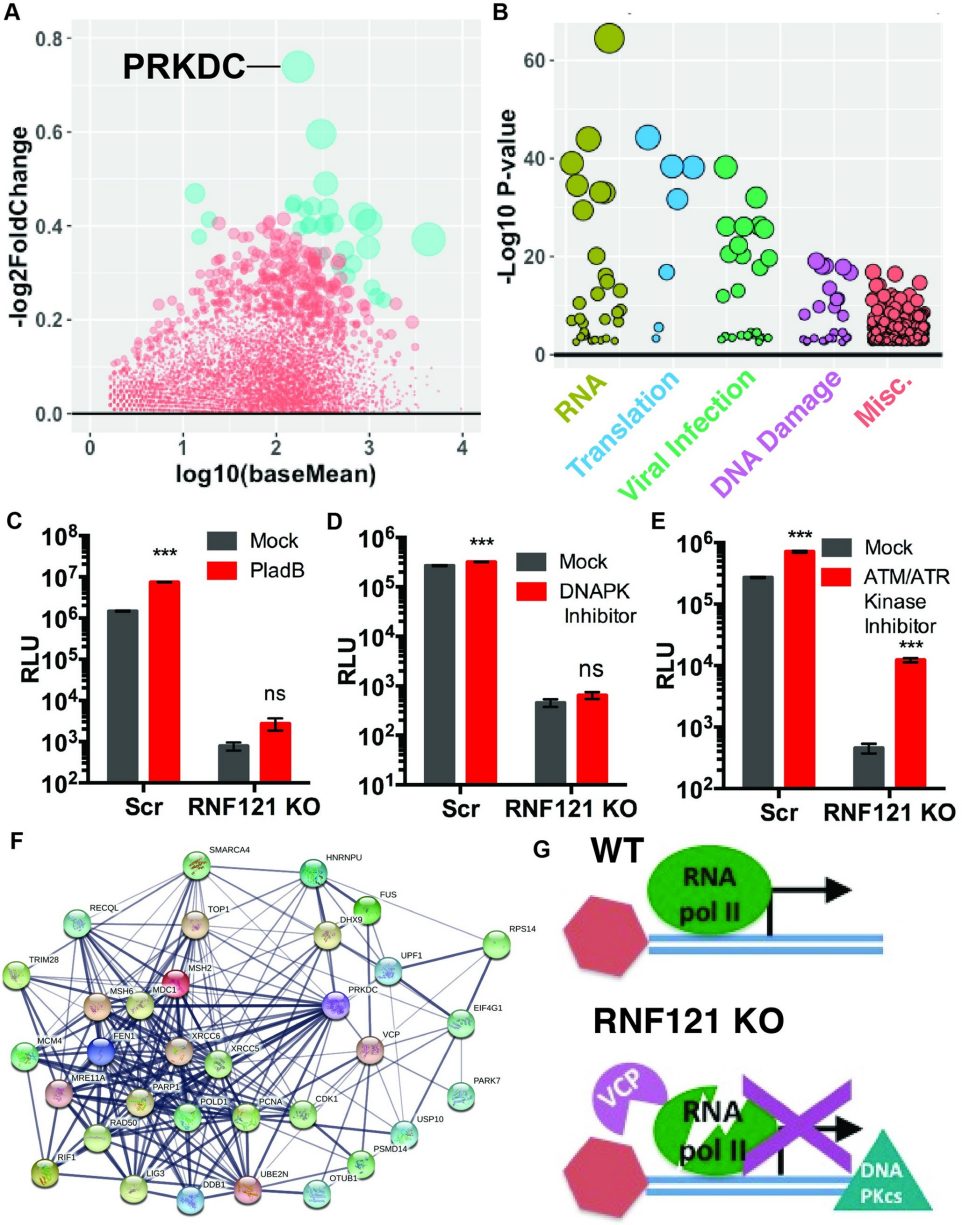
Transcriptomic and proteomic analysis of RNF121 KO cells. A, mRNAs enriched in RNF121 KO, with genes with pAdj<0.05 highlighted in blue. B, Ingenuity pathway analysis of AAV capsid binding partners in RNF121 KO cells showing pathways involved in RNA processes (green), translation (blue), viral infection (lime), DNA damage (purple), and miscellaneous (red). C, U2snRNP inhibitor PladB (20nM) treatment of Scr and RNF121 KO cells. D, DNA-PK inhibitor treatment of Scr and RNF121 KO cells (20uM). E, ATM/ATR Kinase inhibition of Scr and RNF121 KO cells (20uM). F, StringDB interactome of DNA damage and proteasomal machinery in RNF121 KO cells. G, Summary of findings highlighting VCP mediated transcriptional arrest in the absence of RNF121, an essential AAV host factor. A two-tailed unpaired t test was used unless otherwise indicated, with *, p<0.05; **, p<0.01; ***, p<0.005.

In light of the transcriptional arrest of AAV vector genomes in RNF121 KO cells, we carried out transcriptome analysis to identify mRNAs that were deregulated in general. Briefly, we performed RNA seq on Scr and RNF121 KO HEK293, before or after AAV2-Luciferase transduction (10,000 vg/cell). These studies were carried out in biological triplicates and differential gene expression analyzed between samples (Fig 7A, Supplemental Dataset 1). The most significantly upregulated host transcript in RNF121 KO cells was PRKDC, which encodes the catalytic subunit of DNA Protein Kinase (DNA-PKcs). DNA-PKcs has previously been shown to be important for the cellular response to DNA damage. In particular, DNA-PKcs has been shown to recruit VCP/p97 through phosphorylation to DNA damage sites [38]. Moreover, the mechanism by which DNA-PKcs results in transcriptional arrest by reducing the association of RNA pol II at double strand break sites is well known [38,39].

Given the demonstrated importance of the capsid in transcriptional repression of AAV genomes in RNF121 KO cells, we also performed affinity-purification mass spectrometry to better understand capsid binding partners in Scr and RNF121 KO cells. Scr and RNF121 KO cells were transduced with biotinylated AAV2-Luciferase, and a control population of cells was transduced with unlabeled AAV2-Luciferase. These cells prepared in biological triplicate and subject to immunoprecipitation with a biotin antibody, and the efficiency of this pull-down was confirmed as shown in Fig 5B. Scoring of Mass spectromic hits revealed enrichment of known host transcriptional inhibitors associating with the AAV capsid in RNF121 KO cells, including SF3B (Supplemental Dataset 2)[11]. Ingenuity Pathway Analysis of significant hits enriched in RNF121 KO capsids revealed augmented representation of genes associated with RNA processes (green), translation (blue), viral infection (lime), and DNA damage (purple), with some overlap of hits involved in these processes (Fig 7B, Supplemental Dataset 3). For example, while U2 snRNP spliceosome components SF3A3 and SF3B are implicated in multiple RNA processes, these genes were also implicated in viral infection, translational processes, and DNA homologous recombination (Supplemental Dataset 3). Notably, proteomic analysis did reveal enhanced association of both PRKDC and VCP with the AAV capsid in RNF121 KO cells (Supplemental Dataset 2).

To dissect the roles of different pathways highlighted in our transcriptome and proteome results, we performed pharmacological inhibition of notable pathways and assessed the effect on AAV genome expression. U2snRNP inhibition via PladB increased transduction in Scr cells nearly 10 fold, while transduction in RNF121 KO cells was less robustly enhanced (Fig 7C). Inhibition of DNAPKcs did not substantially affect AAV transduction in Scr or RNF121 KO cells (Fig 7D). Conversely, ATM/ATR kinase inhibition resulted in a modest 2.7 fold increase in Scr AAV2 transduction, consistent with previous publications, while RNF121 KO cells experienced a selective, nearly 30 fold increase in transduction with this treatment (Fig 7E) [40]. Given the proven importance of VCP and PRKDC in DNA Repair pathways, we performed a StringDB interactome analysis of factors involved in the enriched DNA repair pathway of our proteomics (p = 2.78e-14), demonstrating multiple DNA damage repair factors with enhanced association with the capsid in RNF121 KO (Fig 7F). Many of these factors have previously characterized roles in inhibiting AAV genome expression [41–43]. Based on these data, we hypothesize that the absence of RNF121 catalyzes enhanced DNA Damage sensing of the AAV capsid genome complex, resulting in VCP mediated transcriptional silencing.

## Discussion

The current study began by exploring the role of RNF121, an E3 ubiquitin ligase that was previously identified as an essential host factor for AAV infection[12]. CRISPR KO of RNF121 causes a robust decrease in AAV genome expression, irrespective of capsid serotype, cell line, transgene, or vector dose. RNF121 does not appear to be essential for early steps in the AAV infectious cycle, including cell surface binding and uptake. Immunofluorescent colocalization with RNF121 suggests that the AAV capsid may associate with this ubiquitin ligase during trafficking through the perinuclear space. However, this phenomenon did not correlate with nuclear entry, uncoating, or second-strand synthesis, which were unaffected. Nevertheless, we considered the possibility that RNF121 might play a role in regulation of the universal AAV receptor, AAVR [12]. However, western blotting demonstrated no difference in AAVR protein levels in RNF121 KO, and transduction of RNF121 KO cells by an AAVR independent serotype (AAV4) yielded a similar transduction deficient phenotype. Collectively, these results suggested that RNF121 might be essential for an infectious event further downstream.

Subsequent efforts were focused on assessing potential mechanisms that might lead to silencing of AAV gene expression. Specifically, H3K27 acetylation and phosphorylated RNA Pol II-ChIP experiments demonstrated that the RNF121 KO mediated defect in AAV transduction is due to transcriptional arrest. We observed loss in transduction efficiency regardless of the different promoter elements incorporated into the AAV genome cassette. In addition, actinomycin D mRNA stability studies further confirmed a defect in mRNA synthesis for AAV vector genomes. Relatedly, a particularly interesting observation in the current study was that RNF121 KO does not alter transgene expression following transfection of plasmid DNA or purified AAV vector genomes in a capsid-independent setting; however, expression from AAV vector genomes associated with the capsid is robustly silenced. Further, addition of capsids *in trans* does not affect transgene expression following transfection of vector genomes, suggesting that repression of AAV genome transcription in RNF121 KO cells occurs only with capsid present *in cis*. Our results are further corroborated by previous studies indicating that the AAV capsid plays a role in the transcription of its genome, where capsid mutants were derived that could traffic effectively to the nucleus and uncoat, but displayed severe transcriptional deficiency mirroring the phenotype observed in RNF121 KO cells [21,44]. These observations together suggest that the transcriptional role of the AAV capsid occurs in *cis*, and that the vector genome must remain associated with the AAV capsid in order to observe the RNF121 related phenotype.

Given the critical role of the AAV capsid outlined above, we carried out *in vitro* ubiquitination assays of the capsid proteins (VP1/2/3) with recombinant RNF121. Unfortunately, we did not observe direct RNF121 mediated ubiquitination of the capsid *in vitro*. Furthermore, immunoprecipitation studies did not reveal a direct interaction between RNF121 and the AAV capsids. While it is plausible that the transient nature of interactions preclude observation of direct RNF121-AAV interactions, we posed the larger question as to whether the E3 ligase activity of RNF121 was essential. To this end, we generated catalytically dead RNF121 mutants, which should demonstrate decreased ligase activity [30]. Attempts to rescue AAV genome expression by transfecting catalytically dead RNF121 constructs demonstrated that the E3 ligase activity of RNF121 is indeed essential for AAV genome expression.

We then adapted a pharmacological approach to interrogate the role of the ubiquitin-proteasome system in AAV transduction in RNF121 KO cells. Inhibition of ubiquitination with a dominant negative ubiquitin construct increases AAV genome expression in Scr, but not RNF121 KO cells. Inhibition of E1 activating enzymes in the ubiquitin pathway increased transduction in Scr and RNF121 KO cells by the same order of magnitude. While proteasome inhibition increases AAV transduction in Scr cells, it does not substantially increase transduction in the context of RNF121 KO. In sharp contrast to these observations, pharmacological inhibition of Valosin containing protein (VCP)/p97 produced essentially restored AAV genome expression in RNF121 KO cells compared to that of control Scr cells. These results suggested that in the absence of RNF121, VCP, a segregase [35], is involved in inactivating the AAV genome transcription machinery.

To further expand on these findings, we applied high-throughput approaches to understand the impact of RNF121 KO on the AAV genome transcription complex. First, transcriptomic profiling of the host genes upregulated in RNF121 KO cells revealed PRKDC, the transcript encoding DNAPKcs, as our lead hit [39]. As further corroboration, we identified both DNAPKcs and VCP as host proteins that were enriched during proteomic analysis of the AAV genome transcription complex in RNF121 KO cells. Interestingly, DNAPKcs is known to phosphorylate VCP recruiting this segregase to double strand breaks [38,45]. Notably, VCP has been shown to function in various chromatin associated degradation processes [36,38,46]. Further, VCP has also been implicated in sites of stalled transcription and RNA Pol II turnover [47]. In addition, DNAPKcs has been shown to cause transcriptional arrest via decreased association of RNA pol II at DNA breaks [39,45]. These observations are consistent with our data showing substantially decreased association of RNA pol II with the AAV genome in RNF121 KO cells. We postulate that VCP functions to inactivate the AAV genome transcription complex in the absence of RNF121. Moreover, our mass spectrometric data identified proteins from the ubiquitin-proteasome pathway such as UBE2N, OTUB1, PARK7, USP10 in addition to VCP associating with the AAV genome transcription complex. These targets warrant further investigation.

Consistent with the notion that DNA damage related processes are involved, we observed enrichment of Rad50, MRE11, XRCC5/6 (Ku86/70), DDB1, PCNA as binding partners of the AAV genome transcription complex in RNF121 KO cells. Several of these host proteins have previously been characterized as host restriction factors that recognize the AAV genome ITRs, while others warrant further investigation [41,42,48]. Furthermore, DNA-PKcs has been demonstrated to localize with and orchestrate the cellular DNA damage response to wild-type AAV replication centers [49,50]. Notably, although Adenovirus type 5 (hAd5) is known to dampen the DNA Damage response through degradation of factors such as Mre11, hAd5 coinfection was not sufficient to rescue wild-type or recombinant AAV genome expression [51]. The failure of hAd5 coinfection or direct pharmacological inhibition of DNAPKcs to rescue the RNF121 KO phenotype, but the ability of an ATM/ATR inhibitor to partially restore AAV gene expression suggests that only certain selective components of the DNA damage machinery might be directly involved.

Overall, we postulate that RNF121, VCP and DNAPKcs are essential components of a regulatory network that controls AAV genome transcription (Fig 7G). Upregulation of DNAPKcs and recruitment of inhibitory host factors such as VCP in the absence of RNF121 appears to inactivate RNA Pol II mediated AAV genome transcription. Because pharmacological inhibition of both VCP and ATM/ATR kinases restores AAV gene expression in RNF121 KO more robustly than in wild-type cells, we believe that DNA damage mediated repression of AAV genomes is augmented when RNF121 is absent. These data are corroborated by enhanced expression of DNA-PKcs, a kinase known to cooperate with ATR and ATM in activating the DNA damage checkpoint, though inhibition of DNA-PKcs alone was not sufficient to rescue AAV transduction in RNF121 KO cells [45,52]. Further studies are warranted to identify and dissect the role of specific RNF121 substrates impacting AAV genome transcription. Nonetheless, our data highlights a novel role for RNF121 in regulating genome transcription for both recombinant and wild type AAVs. Further investigation of the interplay between the ubiquitin-proteasome and DNA damage machinery could shed light on mechanisms underlying transcriptional silencing of AAV genomes with implications for gene therapy.

## Methods

### Plasmid constructs

Guides against RNF121 were designed and ordered as single stranded oligonucleotides from IDT with sequences 5’-GGATCATTGAGAACACGTAT-3’ (Guide 1) and 5’–GTTCCAGAACTCCATAGTAG-3’ (Guide 2). Guides were phosphorylated, annealed, and ligated into BsmBI digested LentiCrisprV2 (a gift from the Feng Zhang lab; Addgene plasmid # 52961) [53]. To generate the RNF121 overexpression construct, RNF121 cDNA was generated by reverse transcription PCR from total RNA isolated from HEK293 cells (adherent human embryonic kidney 293 cell line obtained from the University of North Carolina Vector Core) using Trizol following manufacturer’s protocol (Invitrogen, Carsbad, CA). RNF121a DNA was amplified by PCR (Phusion HF, NEB, Ipswich, MA) and cloned into pCDNA3.1+ using EcoRI and NotI sites. Catalytic mutants were introduced into this backbone via Site Directed Mutagenesis. pRK5-HA-Ubiquitin-KO was a gift from the Ted Dawson lab (Addgene plasmid # 17603) [33]. The TTR enhancer and promoter, as well as the ApoE enhancer and hAAT promoters, were isolated from gblocks (IDT) via restriction enzyme digestion, and cloned along with the MVM intron into an AAV GFP expression cassette.

### Antibodies and chemicals

Mouse anti-actin (ab3280) and mouse anti-KIAA0319L(AAVR) (ab105385) were obtained from Abcam (Cambridge, MA). Goat anti-rabbit-HRP (111-035-003) was obtained from Jackon ImmunoResearch (West Grove, PA). Anti-Ubiquitin antibody P4D1 (sc-8017) was purchased from Santa Cruz Biotechnology (Dallas, TX). Rabbit anti-RNF121 (PA5-61136), Goat anti-Biotin (31852), and Goat anti-mouse-HRP (32430) were obtained from ThermoFisher. Anti-capsid protein antibody B1 [54] was used to blot for capsid protein, while anti-capsid antibody A20 [55] was used for immunofluorescence. MG132 (10012628) was purchased from Cayman Chemical (Ann Arbor, MI). PYR-41 (N2915), DBeQ (SML0031), as well as ATM/ATR (118501) and DNAPK (260960) inhibitors were purchased from Sigma-Aldrich. PladB was a kind gift from the lab of Yasuhiro Ikeda (Mayo Clinic, Rochester, Minnesota).

### Recombinant virus production

Recombinant AAV vectors packaging a chicken β-actin (CBA) promoter driven firefly luciferase cassette, self-complementary AAV (scAAV) vectors packaging a hybrid CBA (CBh) promoter driving GFP, and vectors packaging hAAT and TTR GFP cassettes described above were generated using triple plasmid transfection in HEK293 cells described previously [17]. Viral titers were obtained as previously indicated [17]. Briefly, viral preparation samples were subject to DNAse (10mg/mL) treatment to remove unencapsidated viral genomes. Following inactivation of DNAse with 0.5 M EDTA, samples were subject to Proteinase K digestion to release encapsidated viral genomes. Viral DNA was then measured against vector core standards by quantitative PCR with primers against the ITRs using a Roche Lightcycler 480 (Roche Applied Sciences, Pleasanton, CA). rAAV2-CMV-Luciferase was purchased from the UNC Chapel Hill Vector Core.

Recombinant lentivirus packaging guides against RNF121 or Scr control guides was produced via triple plasmid transfection with psPax2 and VSVG glycoprotein for pseudotyping in HEK293 cells, as previously described [53]. Media supernatant was harvested at 30 and 48 hours post transfection and filtered. Recombinant Ad-GFP vectors were a kind gift from the Xiao Xiao lab at UNC Chapel Hill and wild type human Ad5 was obtained from ATCC (VR-1516).

### Cell lines

Huh7 and U87-MG (human hepatocarcinoma and human glioblastoma cells obtained from the University of North Carolina Lineberger Tissue Culture Facility) and HEK293 (human embryonic kidney cells obtained from the University of North Carolina Vector Core). Cells were maintained in Dulbecco’s Modified Eagle’s Medium (DMEM) supplemented with 10% fetal bovine serum (FBS), 100U/ml penicillin, 100ug/ml streptomycin. Cells were maintained in 5% CO_2_ at 37°C. For generation of stable cell lines, Huh7, HEK293, or U87 were seeded at 1e5-3e5 cells/well in a 6 well plate and incubated with media containing recombinant lentivirus and 8ug/mL polybrene for 10 minutes, followed by spinoculation for 30 minutes at 400g at room temperature. Following spinoculation, cells were incubated for 2–4 hours after which the media was removed and replaced with media containing lentivirus without polybrene. 48 hours post spinoculation cells were selected with puromycin for 7 days, after which clonal lines were generated by serial dilution of cells.

### Luciferase transduction assays

Cells were counted and seeded at equal density (3e4 for Huh7, 1e5 for U87 and HEK293) on 24 well plates and allowed to adhere overnight. Cells were then transduced with AAV-CBA-Luciferase vectors at a dose of 2000 vg/cell unless otherwise indicated. 48 hours post transduction, cells were harvested in Passive Lysis Buffer and lysate combined with Luciferin substrate from Promega (Madison, WI). Luciferase signal was then quantified by a VictorX plate reader from PerkinElmer (Waltham, MA).

### Viral binding, uptake, and trafficking studies

Scr and RNF121 KO Huh7 cells (representing single Huh7 clonal cell lines stably integrated with scrambled guide RNAs or targeted guide RNAs that knockout the RNF121 gene, respectively) were seeded in 12 well plates at a density of 5e4 cells/well 18 hours prior to experiment. Cells were first pre-chilled at 4°C for 30 minutes, and then incubated with rAAV-CBA-Luciferase at 4°C for 1 hr, followed by three washes with ice-cold 1x phosphate buffered saline (1x PBS) to remove unbound virions. 300 uL of ddH20 was then added to each well and cells were subject to three freeze-thaw cycles prior to extraction of total genomic DNA using the IBI Mini Genomic DNA Kit (IBI, Dubuque, IA). Quantification of viral genomes per cell was determined via qPCR of DNA samples with primers against the Luciferase transgene and the host Laminin gene.

For cellular uptake studies, following removal of unbound virions cells were immersed in warm DMEM + 10% FBS 1% P/S and moved to a 37°C incubator to synchronize virus internalization. 1hr post incubation, cells were treated with 300uL/well 0.05% trypsin for 5 minutes to dissociate cell-surface associated virions. Trypsin was quenched with 300uL/well DMEM + 10% FBS 1% P/S, followed by three washes of the cell pellet with cold 1x PBS. Total genomic DNA was then extracted as previously described [17] and vg/cell determined by qPCR.

For trafficking studies, Scr and RNF121 KO Huh7 cells were transduced with rAAV2-luciferase for 18 hours, then cytoplasmic and nuclear fractions were harvested with the NE-PER kit (Thermo Fisher). Fractions were subject to qPCR as previously described to quantify vector genomes.

### Flow cytometry

Cells were washed with 1X PBS, trypsinized, and resuspended in full media. Resuspended cells were then washed twice with cold 1X PBS and stained for viability with Zombie Violet for 30 min at 4 degrees in the dark, per manufacturer protocol (Biolegend). Zombie Violet staining was quenched with IFWB buffer, then cells were fixed with paraformaldehyde and filtered to remove clumps prior to analysis on a CyAn ADP (Beckman Coulter).

### Chromatin immunoprecipitation (ChIP)

ChIP was performed as previously described [56] with HEK293 cells using 10 million cells per replicate and experimental condition. H3K27ac (Abcam, ab4729) and phosphorylated RNA pol2 (Active Motif, 61085) were used. Following ChIP, qPCR was performed with SYBR Green (Roche, 19317900). Enrichment levels were normalized to GAPDH. For each experimental type, 3 biological replicates were analyzed. The following luciferase primers (5’-3) were used: (-) 380 (F’ gcagccattgccttttat, R’ gctccgcacagatttgg); (-)120 (F’ taaccatgttcatgccttct, R’ caaaatgatgagacagcaca); (+) 180 (F’ cctggaacaattgcttttac, R’ gtttcatagcttctgccaac); (+) 550 (F’ atacgattttgtgccagagt, R’ gcagaccagtagatccagag).

### mRNA purification, quantitation, and stability assays

Cells were washed once with 1x PBS then harvested in Trizol Reagent using the manufacturer protocol (Invitrogen, Waltham MA). Following DNAse treatment, cDNA was generated from total RNA via RT-PCR. cDNA samples were then analyzed with qPCR using primers against AAV genomic mRNA and GAPDH housekeeping gene.

For mRNA stability work, 2e5 cells/well were seeded in triplicate into 6-well plates and transduced with 1e5 vg/cell (Scr) or 1e6 vg/cell (RNF121 KO) of the indicated rAAV2 vectors. At 3 days post transduction, media was removed and replaced with fresh media for half an hour. This media was removed and replaced with pre-warmed and equilibrated media containing 5 ug/mL Actinomycin D (Sigma-Aldrich, St. Louis MO). Cells were treated for 30 min, then media was removed and replaced with fresh media. Cells were harvested in Trizol at 0-, 4-, 8-, 12-, and 24-hour time points. RNA was extracted and analyzed by qRT-PCR as described above.

### Confocal fluorescence microscopy

Scr and RNF121 KO cells were seeded on slide covers in 24-well plates at a density of 4e4 cells/well and allowed to adhere overnight. Cells were then transduced with AAV2 for 12 hours, then fixed with 4% paraformaldehyde for 30 minutes and permeabilized with 0.1% Triton X-100 for 30 minutes. Following 30 minutes of blocking with 5% Normal Goat Serum, cells were stained with A20 and RNF121 primaries for one hour, washed 3x with PBS, and then stained with fluorescent secondaries. Cells were subject to 5 minutes staining with DAPI, and then mounted in Prolong Diamond (Invitrogen) and imaged using a Zeiss 710 scanning confocal microscope.

### Capsid biotinylation and immunoprecipitation

AAV capsid was labeled with NHS-PEG4-Biotin (Thermo Fisher #21330) as previously described [19]. Briefly, NHS-PEG4-Biotin was diluted in molecular biology grade water to 2 nM, combined with purified rAAV2-GFP, and incubated for 30 minutes at room temperature. Unbound biotin was removed by buffer exchange using ZebaSpin Desalting Columns (Thermo Fisher #89882).

For immunoprecipitations, Scr and RNF121 KO cells were seeded overnight, and transduced with AAV2 vector with or without biotinylation. 24 hours post transduction, cells were washed with ice cold 1X PBS and harvested in RIPA with 1x Halt Protease Inhibitor (Thermo Fisher) for 30 minutes shaking on ice. Lysates were spun at max speed for 10 minutes at 4 degrees, and supernatant was subject to pre-clearing by 1 hour incubation with 25uL Protein G Magnetic beads (GE) on a mutator at 4 degrees. Goat anti-Biotin was bound to Protein G Magnetic beads for 1 hour at 4 degrees, after which pre-cleared lysate was added to antibody bound beads and rotated overnight at 4 degrees Celsius. Immunoprecipitations were eluted in 10mM DTT and 1X LDS, and analyzed via western blot.

### *In vitro* ubiquitination assay

In vitro ubiquitination reactions were performed using rAAV2-Luc (2.9e12 vg/ml) as a substrate. The ubiquitin thioester/conjugation kits were purchased from Boston Biochem (K-995 and K-980B). Purified recombinant RNF121 was purchased from Abcam (ab163056). The reaction mixture contained 3 μM purified RNF121 and 4.8nM rAAV2-Luc. Concentrations of other components were used following the manufacturer’s instructions. Reactions were performed at 37°C for 2h. Samples were terminated with 4X non-reducing LDS sample buffer (Invitrogen NP0007) supplemented with 10 mM DTT. Samples were boiled at 95°C for 5min, then subjected to SDS-PAGE (NuPAGE 4–12% Bis-Tris Gel) and transferred onto nitrocellulose membrane (ThermoScientific). The signal was visualized with SuperSignal West Femto maximum sensitivity substrate (ThermoScientific) according to the manufacturer’s instructions.

### RNA-seq

Scr and RNF121 KO HEK293 were seeded and transduced with 10,000 vg/cell rAAV2-Luciferase in biological triplicate. Samples were harvested in trizol and subject to phenol-chloroform extraction, and total RNA samples were submitted to Genewiz (Morrisvile, NC) for mRNA library preparation and high-throughput sequencing. Briefly, raw reads were trimmed and aligned with BBMAP, quantified with SubRead, Compared with DeSeq2, and graphed in R version 3.2.4 [57,58].

### Affinity-purification mass spectrometry

Two 15 cm plates of Scr or RNF121 KO HEK293 cells were counted and seeded for each of three biological replicates, and allowed to adhere overnight. Cells were then transduced with biotinylated vector or an unlabeled control at a dose of 2000 vector genomes per cell. 24 hours post transduction, cells were washed with ice cold 1X PBS and harvested in RIPA with 1x Halt Protease Inhibitor (Thermo Fisher), then subject to immunoprecipitation as described above. In lieu of elution with LDS and DTT, beads were washed three times with ice cold 1X PBS, then subject to on-bead digestion and affinity purification mass spectrometric analysis with the UNC Proteomics Core.

Raw spectral count data was subject to analysis with the MiST (mass spectrometry interaction statistics) system, as previously described [59,60]. Targets were analyzed for pathway representation with Ingenuity Pathway Analysis (Qiagen), and graphed with R version 3.2.4. Mass spectrometric binding partners represented in the DNA Repair pathway and enriched in RNF121 KO capsids were mapped using StringDB [61].

### Statistical analysis

All data are expressed as mean with error bars representing standard error. A two-tailed unpaired student t test was used or two-way ANOVA where indicated, calculated with GraphPad Prism Version 6. *p* values less than 0.05 were considered significant. Asterisks are used to denote *p* values as follows: *, *p*<0.05; **, *p*<0.01; ***, *p*<0.005.

## Supporting information

S1 Fig High-throughput sequencing data of genomes from RNF121 KO cell lines. A, Summary of genotypes of Scr control and RNF121 KO clonal cell lines. Mutation rates of target indel region in Scr (top) and RNF121 KO (bottom) cells in HEK293 (B), Huh7 (C), and U87 (D).
(TIF)

S2 Fig AAV vector titers from RNF121 KO HEK293 cells. A, Titers from preparations of AAV2-Luciferase from Scr and RNF121 KO HEK293 cells. B, Transduction of wtHEK293 with AAV2-Luciferase produced by Scr and RNF121 KO HEK293 cells.
(TIF)

S3 Fig RNA Pol II and H3K27ac association with GAPDH. ChIP Enrichment of serine 5 phosphorylated RNA pol II (A)and H3K27ac (B) with GAPDH gene in Scr and RNF121 KO HEK293 cells.
(TIF)

S1 Data RNASeq of Scr versus RNF121 KO AAV2 transduction. (XLSX)

S2 Data Mass spectrometric analysis of Scr and RNF121 KO AAV capsid binding partners. (XLSX)

S3 Data Ingenuity pathway analysis of mass spectrometric hits for capsid binding partners. (XLSX)
