# Supplementary figures and images for: Ring finger protein 121 is a potent regulator of adeno-associated viral genome transcription
Source: PLoS Pathog. 2019 Aug 6;15(8):e1007988. doi: 10.1371/journal.ppat.1007988 (PMC6697353; doi:10.1371/journal.ppat.1007988)

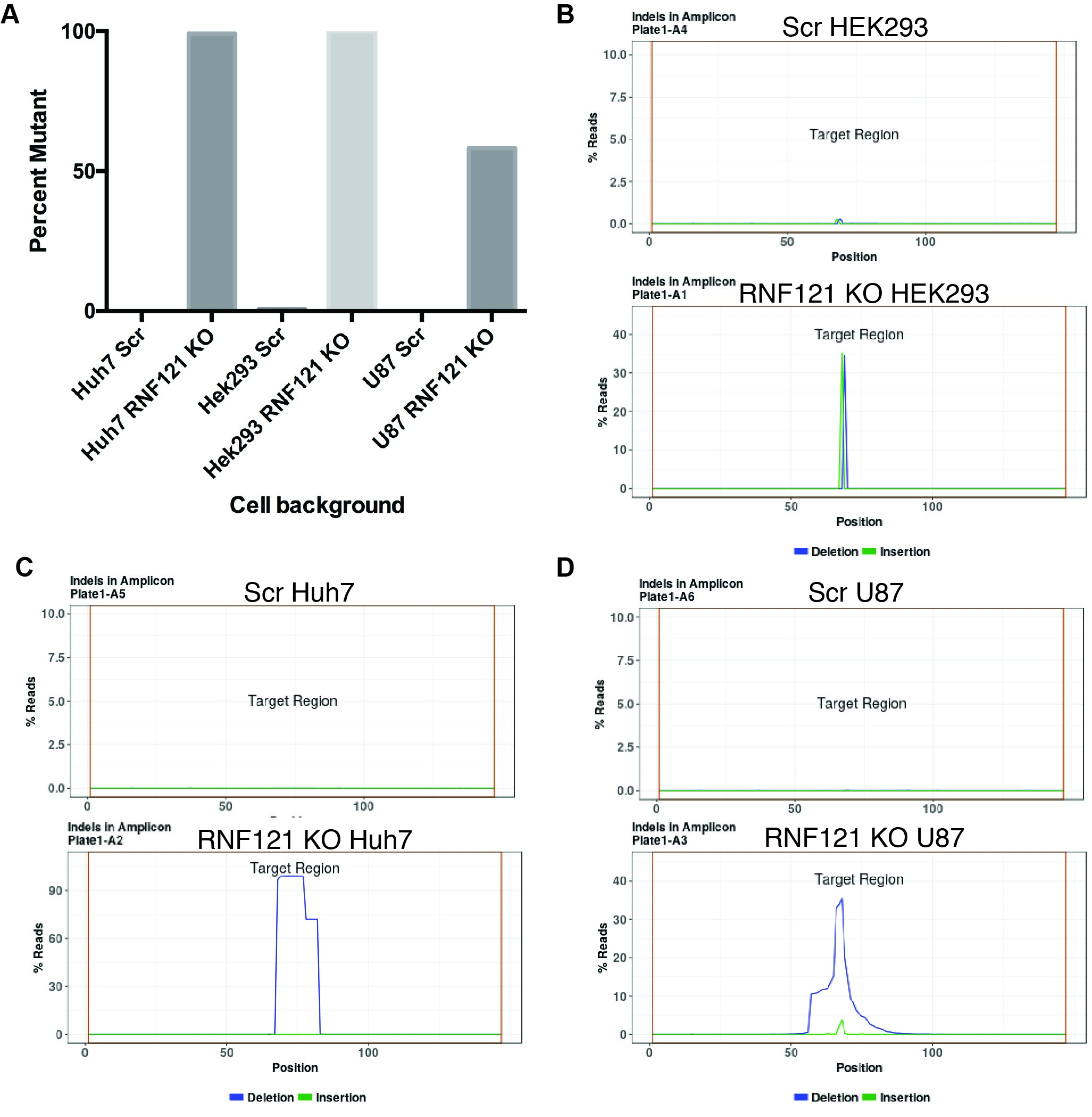

Supplement: S1 Fig — A, Summary of genotypes of Scr control and RNF121 KO clonal cell lines. Mutation rates of target indel region in Scr (top) and RNF121 KO (bottom) cells in HEK293 (B), Huh7 (C), and U87 (D). (TIF) [file ppat.1007988.s001.tif]

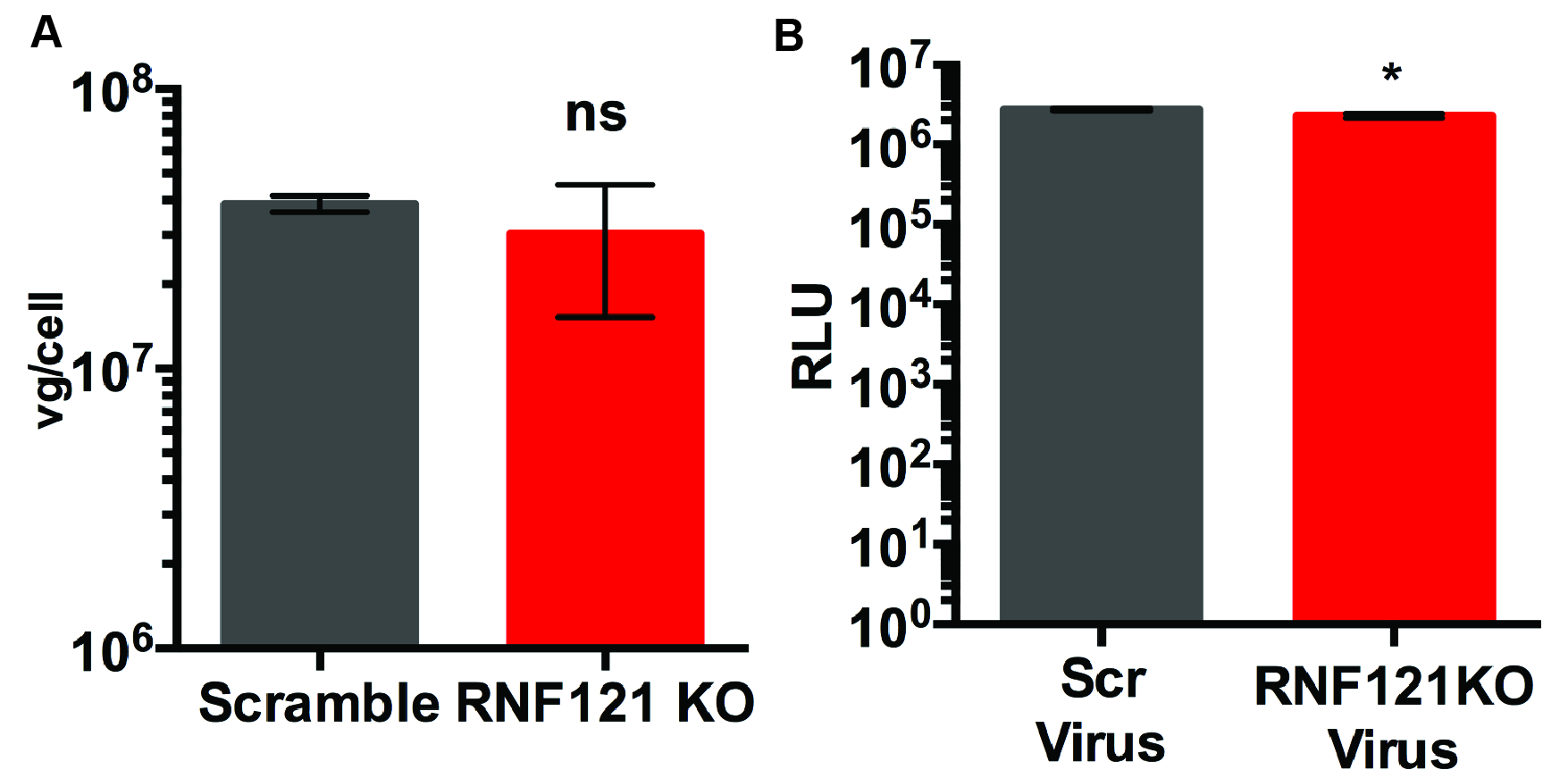

Supplement: S2 Fig — A, Titers from preparations of AAV2-Luciferase from Scr and RNF121 KO HEK293 cells. B, Transduction of wtHEK293 with AAV2-Luciferase produced by Scr and RNF121 KO HEK293 cells. (TIF) [file ppat.1007988.s002.tif]

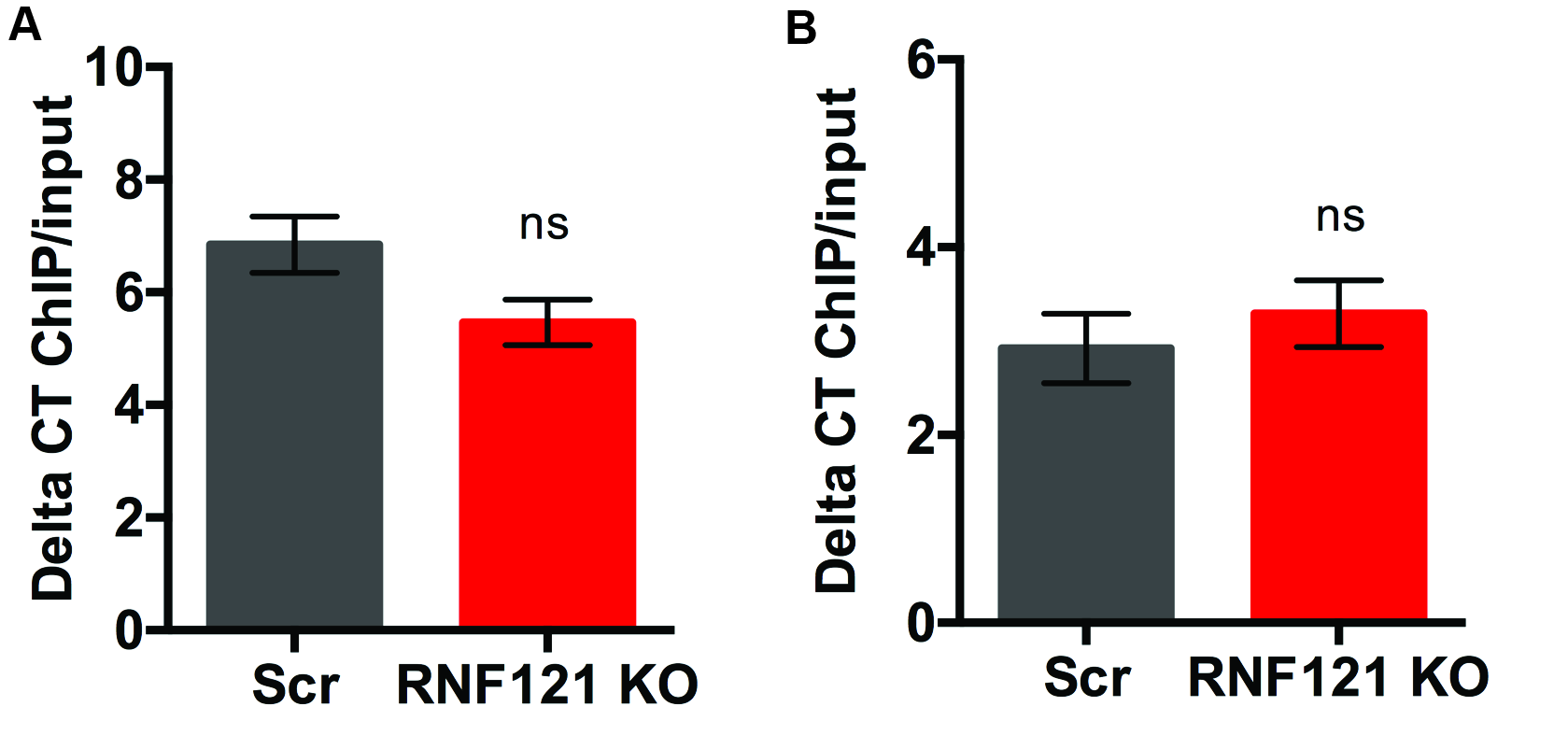

Supplement: S3 Fig — ChIP Enrichment of serine 5 phosphorylated RNA pol II (A)and H3K27ac (B) with GAPDH gene in Scr and RNF121 KO HEK293 cells. (TIF) [file ppat.1007988.s003.tif]
